# The Book World of Medicine and Science

**Published:** 1906-11-17

**Authors:** 


### THE BOOK WORLD OF MEDICINE AND SCIENCE.

Davos as a Health Resort: a Handbook by Various Authors, with an Introduction by W. R. Huggard, M.A., M.D. (Davos Printing Company. 1906. Pp. 316. Illustrations 50.)

This volume, which comes to us franked by Dr. Huggard, the author of "Climatic Treatment and Balneology," claims to be a handbook to the famous health resort situated in the heart of the Rhætian Highlands, and a perusal of the work has convinced us that the claim is well sustained. Most of the articles are by men who are resident in Davos and therefore well acquainted with the place in all its aspects. Eleven of the thirteen articles have been translated into English by Mr. Locket, the Editor of the *Davos Courier*. The chapters most interesting to the medical reader are "The Davos Climate," by Dr. Muehle; "The Physiology of the Davos Climate," by Dr. van Voornveld, Director of the Dutch Sanatorium; "Indications and Contra-Indications for the High Mountains," by Dr. Philippi, Director of the New Sanatorium; "The Treatment of Phthisis in the High Mountains," by Dr. Nienhaus, Director of the Basle Sanatorium, Davos-Dorf; "The Fear of Infection at Davos," by Dr. Jessen of Davos; and "Practical Hints for Sending Patients to Davos," by Dr. Turban, Director of the Sanatorium at Davos-Platz. The reputation of Davos in the treatment of phthisis, and we may add the reputation of all other mountain resorts, is owing to Dr. Sprengel, who as early as 1853 saw some of the wonderful results obtainable when he was district doctor in the neighbourhood; and in 1862 Dr. Meyer-Ahrens visited Davos, when Sprengel drew his attention to the facts. In February 1865 two consumptives appeared on the scene, and were benefited by residence in the High Mountains. From this small beginning the place grew in favour annually until the year 1905, when, we learn from Dean Hauri's paper, Davos was visited by 20,042 persons—of course not nearly all invalids—and of this total 2,379 were English. Naturally this growing influx of patients and visitors had the result of enormously extending the hotel and sanatorium accommodation. Now it is possible to live in the smaller boarding-houses at from 4.50 to 7.50 francs a day, and the scale rises to 20 francs in some of larger and better Sanatoria. What is of great importance is that the drainage is good. One of the authors states that "the sanitary conditions in Davos are much in advance of anything to be found in Switzerland." As to the class of patients which should be sent to Davos it is certain, as Dr. Phillipi puts it, "that the more recent and the slighter the lung affection, the more certain and more complete the success to be looked for"; and this is precisely what holds good everywhere else. Nevertheless the extent of the diseased lung need not of itself preclude treatment at Davos; and as the practitioner has often to decide the question he can hardly do better than remember what Dr. Huggard has said in the forty-fourth chapter of his work, "that the cold, dry climate characteristic of the Alpine resorts is suitable for persons of robust constitution apart from the local disease," and also for patients who though not robust if their "organs of nutrition and oxidation are sufficiently sound functionally to do much more work than they have been accustomed to do at home." Having decided on the high treatment it is worse than useless to await the arrival of any particular month or season to begin that treatment. For the satisfaction of those healthy people who may wish to accompany rich relatives to Davos, we may say that Dr. Jessen assures us there is little or no fear of infection; but rather that should any predisposition to phthisis exist it is likely to be lost there. Other chapters which we have not space to notice relate to the "English Colony at Davos," the "Geology of the Neighbourhood," "The Flora of Davos," "The Davos Peasantry," "Summer in the High Mountains," and "Sport and Phthisis."

The Principles and Exact Conditions to be Observed in the Artificial Feeding of Infants. By W. B. Cheadle, M.D., F.R.C.P. Sixth edition. Edited and revised by F. J. Poynton, M.D., F.R.C.P. (London: Smith, Elder and Company, 1906. Pp. xvi. and 274.)

There is little need to describe or criticise a book so well known as the above. Dr. Cheadle's sound clinical methods and capacity for plain description have happily been applied to the difficult and perplexing problems arising in connection with the artificial feeding of infants, and the medical profession has recognised the value of the result by demanding a sixth edition of his book. Dr. Poynton has edited this latest issue and has brought the book fully up to date. This is of course an advantage and to be welcomed. But substantially the book maintains its original character, and we gratefully recognise it as one of the best practical guides on the subject of which it treats.

